# Diagnosis and management of mast cell activation syndrome (MCAS) in Canada: a practical approach

**DOI:** 10.1186/s13223-025-00998-9

**Published:** 2025-11-21

**Authors:** Erika Lee, Matthieu Picard

**Affiliations:** 1 Division of Allergy and Clinical Immunology, Department of Medicine, University of Toronto, Toronto, ON Canada; 2 Division of Respirology, Univeristy Health Network, Toronto, ON Canada; 3 Drug Allergy Clinic, Sunnybrook Health Sciences Centre, Toronto, ON Canada; 4 Division of Allergy and Clinical Immunology, Department of Medicine, Hôpital Maisonneuve-Rosement, QC Montreal, Canada; 5 Centre de Recherche de l’Hôpital Maisonneuve-Rosemont (CRHMR), Montreal, Canada; 6 Université de Montréal, QC Montreal, Canada

**Keywords:** Mast cell activation syndrome, Diagnosis, Treatment, Management, Anaphylaxis, Idiopathic, Mastocytosis, Monoclonal, Urine metabolites, Tryptase

## Abstract

An increasing number of patients are presenting to allergists with concerns about mast cell activation syndrome (MCAS), often in the context of persistent, unexplained, multisystem symptoms. This review aims provide a practical, stepwise approach to the diagnosis and management of MCAS, based on the consensus criteria established by the European Competence Network on Mastocytosis—American Initiative on Mast Cell Diseases, an international consortium of leading experts in mast cell disorders endorsed by major scientific organizations. The first step is to evaluate whether the clinical presentation is consistent with MCAS, recognizing that the prototypical presentation is idiopathic anaphylaxis. Symptoms should be severe, episodic, typical of mast cell activation, and involve at least two organ systems. The next step is to exclude secondary causes of mast cell activation, particularly cofactor-dependent food allergy and nonsteroidal anti-inflammatory drug hypersensitivity. Objective evidence of mast cell activation must then be obtained, preferably by identifying an acute increase in serum tryptase (on a sample drawn within four hours of an episode) compared to baseline. Alternatively, urinary metabolites of mast cell mediators can be assessed by comparing baseline values with those obtained 3–6 h post-event. Importantly,elevated baseline values in serum tryptase or urinary metabolites are not diagnostic of MCAS, nor do normal values exclude the diagnosis. In patients with idiopathic anaphylaxis, evaluation for a clonal mast cell disorder is recommended. This includes measuring baseline serum tryptase, testing for the KIT p.D816V mutation in peripheral blood (using high-sensitivity assays, if available), and calculating a mast cell clonality prediction score. A bone marrow biopsy should be considered for those with a high probability of mast cell clonality. Management includes instructing patients to treat acute episodes with an epinephrine auto-injector, particularly when anaphylaxis criteria are met. For patients with recurrent episodes, prophylactic therapy may be initiated, starting with H1-antihistamines and stepping-up as needed. While most patients have a favourable clinical course, some may require multiple medications to prevent or attenuate episodes. Future research should focus on validating and refining diagnostic and therapeutic strategies. In clinical practice, expanding access to key diagnostic tools—such as urinary mediator assays, sensitive KIT mutation testing, and tryptase genotyping—would facilitate and improve care of those patients.

## Introduction

Mast cell activation syndrome (MCAS) is a heterogenous condition characterized by acute, episodic, and recurrent symptoms typical of mast cell activation affecting at least two organ systems [1–5]. Although this entity has been discussed for decades in the medical literature, it was only in the 2000s that the term MCAS gained wider usage [1, 6]. Initially a well-defined condition, it was soon linked to numerous nonspecific symptoms despite a lack of solid evidence that it could provide a satisfactory explanation for such patients [1]. 

In an effort to establish an evidence-based approach to diagnose MCAS and to avoid overdiagnosis, Akin et al. proposed in 2010 the first diagnostic criteria [1]. They also proposed a classification of mast cell activation disorders: primary (if a clonal mast cell disorder is identified), secondary (if a well-defined condition is identified as the cause of mast cell activation), and idiopathic [1]. These diagnostic criteria and classification were largely endorsed by an international group of experts in 2012 and have slightly evolved since then with the addition of two other MCAS categories: combined or mixed (patients meeting criteria for primary and secondary MCAS) and hereditary alpha-tryptasemia(HαT)-associated MCAS (patients with either primary, secondary or idiopathic MCAS and HαT) [3]. These criteria are now being referred to as the European Competence Network on Mastocytosis – American Initiative on Mast Cell Diseases (ECNM-AIM) consortium criteria [2, 3, 5, 7]. This international consortium of leading experts in mast cell disorders is endorsed by major scientific organizations, such as the American Academy of Allergy, Asthma and Immunology [3, 8].

In parallel, a separate group of medical professionals have entertained much broader diagnostic criteria for MCAS, which they describe as an extraordinarily complex and variable disease caused by chronic aberrant mast cell activation affecting up to 17% of the population [9]. In short, patients reporting any constellation of symptoms among a very long list (which are not better accounted for by another condition) and with at least a partial response to anti-mediator treatment would meet criteria for MCAS [9]. These criteria have been criticized for their lack of specificity and of supporting evidence to link such a broad range of symptoms to chronic mast cell activation [4, 7, 10–12]. Whereas some patients with MCAS may report a wide array of symptoms such as headache, fatigue, brain fog, difficulty concentrating, and poor memory—in addition to recurrent, circumscribed episodes of typical mast cell activation (e.g.: anaphylaxis)—the cause of these chronic symptoms remain elusive and their response to anti-mediator treatment is variable [11]. Although several authors have postulated an association between MCAS and either postural orthostatic tachycardia syndrome (POTS), hypermobile Ehlers-Danlos syndrome (hEDS), or post-acute sequelae of COVID-19—also known as long COVID—,these associations have never been demonstrated in patients unambiguously meeting ECNM-AIM consortium criteria [8, 13–18].

This review uses the diagnostic criteria of the ECNM-AIM consortium and provides a practical roadmap, specifically tailored for Canadian allergists, to diagnose and manage MCAS while assessing for the co-existence of a clonal mast cell disorder (CMCD) and ruling out key secondary causes of mast cell activation (MCA). Although MCAS can be diagnosed in children, to our knowledge, there is no published cohort of pediatric MCAS patients and the approach presented in this review is mainly based on findings in adults.

## Diagnosis

To diagnose MCAS, all three ECNM-AIM consortium criteria have to be met [3]:

1. Typical clinical signs of severe, recurrent (episodic) systemic MCA are present (often in the form of anaphylaxis) (definition of systemic: involving at least 2 organ systems).
2. Involvement of mast cells (MCs) is documented by biochemical studies: preferred marker: increase in serum tryptase level from the individual’s baseline to 120% + 2 ng/mL. Other MC-derived biomarkers of MC activation (recommended: 24-h or spot urinary histamine metabolites or PGD_2_ metabolites) may also be used, but are less specific compared with the increase in serum tryptase level. In addition, to date, no diagnostic thresholds for the increase in these urinary biomarkers have been defined and validated. Nevertheless, these alternative markers are recommended when the tryptase test is not available or its result is inconclusive. A proposed diagnostic threshold for histamine or PGD2 metabolites is > 200% of the individual’s baseline (increase by >  + 100%) provided that the test result is above the normal range for the assay.
3. Response of symptoms to therapy with MC-stabilizing agents, drugs directed against MC mediator production, or drugs blocking mediator release or the effects of MC-derived mediators.

**Fig. 1:**
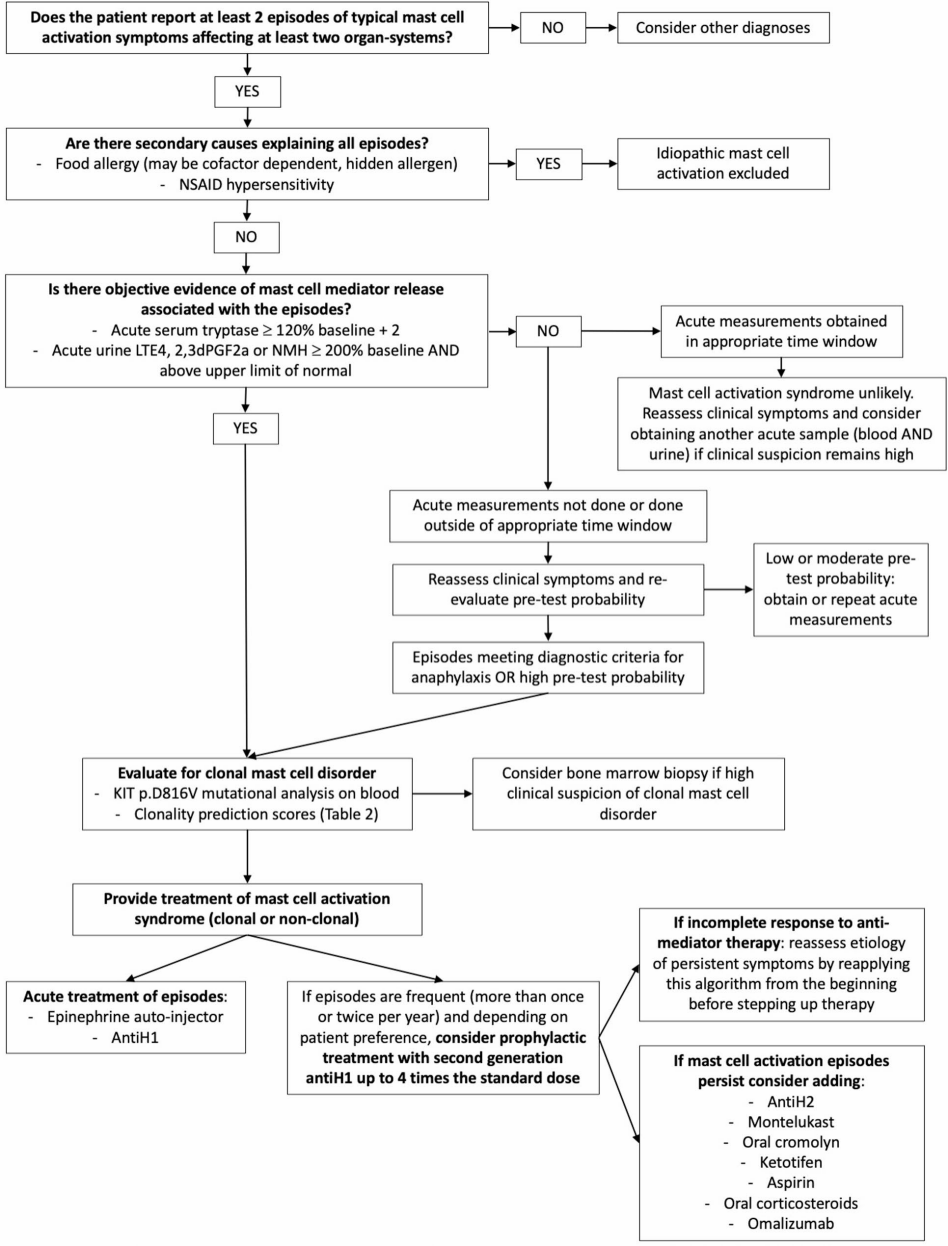
Stepwise practical approach to diagnose and treat MCAS

In practice, we suggest a stepwise approach to diagnose MCAS starting with a thorough assessment of the episodic symptoms to judge if they qualify as typical symptoms or signs of severe and recurrent systemic MCA (Fig. 1 and Table 1). At that time and depending on clinical presentation, other diagnoses could be considered, such as chronic spontaneous urticaria (CSU),or carcinoid syndrome, although the clinical recognition of anaphylaxis is often relatively straightforward for experienced allergists [6]. For example, patients with CSU without MCAS do not exhibit acute episodes of mast cell activation involving two 2 organ systems. Then, well-defined secondary causes of MCA should be explored (e.g. food allergy, nonsteroidal anti-inflammatory drugs (NSAID) hypersensitivity), and if none is identified, biochemical confirmation of MCA should be sought (Table 2). Investigations for primary (clonal) mast cell disorders should also be performed given their prevalence in MCAS patients [19–21]. Treatment can be offered following initial evaluation when clinical suspicion of MCAS is high, especially for patients with severe symptoms, and response assessed on follow-up visits.

**Table 1:** Typical clinical manifestations of MCAS and selected conditions and symptoms not suggestive of MCAS

*Typical clinical manifestations*: sudden onset episodes with symptoms typical of MCA (see list below), affecting at least two organ systems, and resolving within several hours (often meeting anaphylaxis criteria)	
Skin	pruritus (commonly diffuse but that may start in the palms and/or soles), flushing, urticaria, or angioedema
Cardiovascular	objective evidence or symptoms suggestive of hypotension such as dizziness, confusion, pre-syncope or syncope
Gastro-intestinal	abdominal cramping, diarrhea or vomiting
Respiratory	dyspnea, or oxygen desaturation
Oculo-nasal	severe nasal congestion, watery rhinorrhea, sneezing, or conjunctival injection
*Selected conditions and symptoms not suggestive of MCAS* [7]*:*	
Postural orthostatic tachycardia syndrome (POTS), joint hypermobility or hypermobile Ehlers-Danlos syndrome (hEDS), long COVID, idiopathic environmental intolerance (multiple chemical sensitivity), irritable bowel syndrome or multiple food intolerance, bloating, fatigue, headache, brain fog, joint pain, non-episodic symptoms (e.g. chronic urticaria), symptoms isolated to one organ-system (e.g.: skin flushing)	

MCA: mast cell activation

**Table 2:** Suggested investigations in patients with suspected MCAS

Investigations	At baseline	During an acute episode
Serum tryptase	If above 8 ng/ml, consider: • Tryptase genotyping • Creatinine • Complete blood count • KIT p.D816V mutational analysis on blood + Examine for skin lesions of mastocytosis	Sample within 4 h of symptom onset Significant elevation if level is at least 120% + 2 compared to baseline Interpret in relation to clinical symptoms and timing of sampling
Urinary metabolites of mast cell mediators*: N-methylhistamine 2,3-dinor 11β-Prostaglandin F2α Leukotriene E4	Random urine sample or 24-h urine collection If above upper limit of normal, consider bone marrow biopsy in light of other investigations and clinical presentation	Urine sample around 3–6 h after symptom onset Significant elevation if level at least 200% compared to baseline AND above upper limit of normal
KIT p.D816V mutational analysis on blood	Preferred technique (allele-specific or digital droplet PCR with sensitivity of at least 0.01%)* If positive, consider bone marrow biopsy	NA

^*^ Test may not be available in your region of practice. Consider the possibility of a send-out (see text)

NA: not applicable

- Typical episodic symptoms of mast cell activation

To consider a diagnosis of MCAS, the patient needs to report at least two sudden onset episodes of typical MCA symptoms affecting at least two organ systems and resolving within several hours [3]. Typical MCA symptoms are the same as those found in systemic IgE-mediated reactions or anaphylaxis of any cause [22]. In fact, idiopathic anaphylaxis (IA) is the prototypical clinical presentation of MCAS [7, 23]. However, some MCAS patients have typical systemic symptoms of MCA affecting two organ systems that do not qualify for anaphylaxis given insufficient severity (e.g.: diffuse skin symptoms and naso-ocular symptoms or diffuse skin symptoms with moderate abdominal cramping and diarrhea) [7].

Most commonly, patients will report abrupt onset of skin symptoms consisting of pruritus (commonly diffuse but that may start in the palms and/or soles), flushing, urticaria, or angioedema [19]. A majority of patients will report accompanying gastro-intestinal (GI) symptoms such as abdominal cramping, acute diarrhea or vomiting [19]. Cardiovascular involvement is also frequent with either objective evidence or symptoms suggestive of hypotension such as dizziness, confusion, pre-syncope or syncope [19]. Although less frequent than skin, cardiovascular or GI symptoms, respiratory symptoms may also be reported, such as dyspnea, oxygen desaturation, severe nasal congestion, watery rhinorrhea, or sneezing [7, 19]. Occasionnaly, conjunctival injection may be seen [7]. A sensation of throat tightness or closure can be reported by some patients but is very rarely the main symptom and never the sole symptom of a systemic MCA episode. Symptoms occur abruptly and resolve with or without treatment within several hours. Importantly, they should not be mild or chronic or waxing and waning without clearly defined episodes [4, 7]. Episodes can vary in severity over time but are often remarkably similar to one another [24]. Finally, patients may report moderate to severe fatigue following anaphylaxis typically lasting ≤ 24 h [6].

b) Evaluating secondary causes of mast cell activation

Because symptoms alone cannot differentiate between a spontaneous systemic MCA event and one triggered by a specific cause, the circumstances surrounding each episode should be thoroughly assessed. Some patients will report episodes waking them up in the middle of the night with no exposure to food or medication for at least 6 h thereby ruling out a secondary cause of mast cell activation [25]. In others, episodes are mostly or solely provoked by exercise, high ambient temperature or stressful events [6, 26]. However, when episodes occur solely, or mostly, after meals, typically within 1 or 2 h, it should prompt a thorough investigation for an IgE-mediated food allergy [27]. In this scenario, rather than panel testing for food allergies, which may yield false-positive results, a more effective approach may be to perform skin testing with fresh foods from the meal preceding the mast cell activation episode [28]. If skin testing is negative or inconclusive, a challenge with the full meal—ideally including cofactors such as alcohol, NSAIDs, or exercise, if they were present—can be conducted [29, 30]. If wheat was eaten before most or all episodes, and even if tolerated at other times, skin testing to gluten flour (which is enriched in wheat protein compared to regular flour) and/or specific IgE testing to wheat and Tri a 19 should be considered given the possibility of cofactor-dependent wheat allergy, which can masquerade as IA [31, 32]. In patients sensitized to non-specific lipid transfer proteins in fruits and vegetables, systemic MCA may occur only in the presence of cofactors [33]. Skin testing to the fresh food can help rule out this possibility but a positive result does not prove causality and a food challenge, with or without cofactor (NSAIDs, exercise, alcohol), should be considered [33].

Unrecognized or hidden allergens can be particularly challenging to identify although they appear to be an uncommon cause of unexplained MCA [27]. One of the authors evaluated a patient, known to have an IgE-mediated buckwheat allergy, with recurrent post-prandial MCA episodes for almost a year before the patient realized she was using a pepper seasoning containing buckwheat. Although alpha-gal syndrome is often mentioned in the differential diagnosis of MCAS, this entity is rare in Canada given the geographical distribution of the lone star tick [27, 34]. If this diagnosis is suspected, alpha-gal specific IgE can be ordered.

NSAIDs can be responsible for systemic MCA episodes with onset several hours after intake, or act as a cofactor for food allergies [25, 29, 35]. Therefore, NSAID intake within 6 h of a mast cell activation episode should be actively sought and when in doubt, a drug challenge should be considered.

c) Obtaining objective evidence of mast cell activation

Obtaining objective evidence of mast cell activation, through measurement of serum tryptase or urine metabolites of mast cell mediators in relation to episodic symptoms, is required for confirming a diagnosis of MCAS [3]. In contrast, a diagnosis of IA can be made solely on clinical grounds although in practice we would try obtaining objective evidence of MCA [23]. Acute measurement of serum tryptase or urine metabolites of mast cell mediators is especially important when symptoms do not meet diagnostic criteria for anaphylaxis or are not entirely typical of MCA [3, 7]. Also, when some episodic symptoms persist under anti-mediator therapy, it can be important to ascertain that they are truly caused by systemic MCA before stepping up therapy.

Serum tryptase is the most specific mast cell mediator and also the most validated to confirm MCA [3, 5]. It should be measured at baseline and within 4 h of an episode [3, 5, 7]. Because of its short half-life, its sensitivity to detect a MCA episode decreases rapidly with time elapsed since symptom onset [7, 8]. Therefore, even when measured within 4 h, it could fail to show a significant elevation compared to baseline, which is defined by expert consensus as a value at least 1.2 × baseline tryptase + 2 ng/mL (120% + 2 formula) [3, 5]. Baseline serum tryptase can vary over time—sometimes by more than 120% + 2 ng/mL—without clinical evidence of MCA, especially in patients with an elevated baseline level [36]. The acute tryptase value should therefore always be interpreted in relation to symptoms (typical versus atypical) and time elapsed since symptom onset [37]. Mateja et al. have reported that an acute increase of at least 68.5% over baseline tryptase (acute/baseline ratio: 1.685) had a sensitivity and specificity of 95% for anaphylaxis [36]. The ratio could also be adjusted based on pre-test probability of anaphylaxis (high probability: 1.374 and low probability: 1.868) [36]. However, these ratios still require external validation. Importantly, an elevated baseline serum tryptase, present in around 6% of the population, should never be interpreted as a sign of mast cell activation (see later discussion on HαT) [5, 7]. In practice, it can be helpful to ask the patient to keep a requisition form for serum tryptase with the epinephrine auto-injector so that it is readily available when an episode occurs and the requisition can be shown upon arrival to the emergency room.

Given the complexity of obtaining a timely sample of serum tryptase during an acute episode, urine metabolites of mast cell mediators can be measured at baseline on a random, or 24-h, urine sample and compared with their levels on a spot urine sample obtained around 3–6 h after an episode [3, 8, 38–40]. The most commonly used metabolites are: N-methylhistamine (NMH), 2,3-dinor 11β-Prostaglandin F2α (2,3dPF2α), and leukotriene E4 (LTE4). However, they are less specific than serum tryptase for MCA, the optimal timing for their measurement is not as well established and the cut-off used for confirming MCA varies between authors [3, 41]. According to the most recent diagnostic criteria for MCAS, a 200% increase over baseline levels and that is above the normal range of the assay could be considered objective evidence of MCA [3]. Following anaphylaxis, LTE4 and 2,3dPGF2α often increase several folds compared to baseline while the increase in NMH is often less pronounced and sometimes absent [41, 42]. A recent study has suggested that an acute increase as low as 30% above baseline in any urine metabolite could correlate with an acute increase of 20% + 2 ng/mL in serum tryptase [41]. However, the specificity of this lower threshold for systemic mast cell activation remains to be studied. To our knowledge, measurement of those 3 mediators on a random urine sample is not currently available in any Canadian laboratory but a send-out can be arranged to a specialized laboratory [43–45]. In practice, patients can have baseline urine metabolites measured on a random urine sample at any time and can be asked to collect a urine sample around 3 h after a suspected MCA episode at home. The specimen should then be kept in the refrigerator and brought to a nearby hospital with a pre-filled requisition form as soon as possible so that a send-out can be arranged by the hospital laboratory.

d) Evaluating clonal mast cell disorders

CMCD, such as monoclonal mast cell activation syndrome (MMAS) and systemic mastocytosis (SM), may present with recurrent IA, or severe hymenoptera sting anaphylaxis, especially if characterized by hypotension and absence of skin symptoms [3, 19, 37, 46–49]. Male patients and those with an elevated baseline serum tryptase appear at higher risk [46, 50–52]. Prediction scores such as Spanish Network on Mastocytosis (REMA), Karolinska (same as REMA but using lower serum tryptase cut-off values) and National Institute of Health Idiopathic Clonal Anaphylaxis Analysis Score (NICAS) can be helpful in predicting which patients are at higher risk of having an underlying CMCD and may therefore require a bone marrow biopsy (Table 3) [46, 50–52]. The scores remain predictive of CMCD even if HαT is diagnosed and accounts for the elevated baseline tryptase [53].

**Table 3:** Prediction scores for clonal mast cell disorders in patients with recurrent idiopathic anaphylaxis

Characteristics	REMA	Karolinska	NICAS
Male	+ 1	+ 1	+ 1
Female	-1	-1	-1
Baseline serum tryptase	< 15 ng/mL: -1 15-25 ng/mL: 0 > 25 ng/mL: + 2	< 11.4 ng/mL: -1 11.4-20 ng/mL: 0 > 20 ng/mL: + 2	< 11.4 ng/mL: -1 > 11.4 ng/mL: + 1
Anaphylaxis symptoms:			
- Syncope	+ 3 (or presyncope)	+ 3 (or presyncope)	+ 3
- Pruritis, hives or angioedema	Present: -2 Absent: + 1	Present: -2 Absent: + 1	NA
- Urticaria	NA	NA	Present: + 1
- Flushing	NA	NA	Present: -1
- Angioedema	NA	NA	Absent: -1
KIT p.D816V allele specific quantitative PCR mutational analysis on blood	Present: indicative of mast cell clonality regardless of score*	Present: indicative of mast cell clonality regardless of score*	Absent: -1 Present: + 3
Score interpretation	≥ 2: predictive of mast cell clonality: consider bone marrow biopsy		

^*^A positive KIT D816V mutational analysis on blood is usually diagnostic of mast cell clonality but, alone, lacks sensitivity, especially for MMAS[19, 53]

HαT should be suspected in any patient with a baseline serum tryptase value above 8 ng/mL since it is the leading cause of hypertryptasemia, affecting around 6% of the population [37, 54, 55]. Genetic testing for HαT involves TPSAB1 copy number analysis coupled with TPSB2 genotyping and is offered by some specialized laboratories [56, 57]. To our knowledge, this test is not offered by any Canadian laboratory thus requiring a send-out. Alternatively, patients who are willing to pay for the test themselves can order it online, perform the buccal swab, and send it back for analysis along with a completed requisition form by their physician [57]. If genetic testing for HαT is not feasible, it may be reasonable to follow tryptase over time after exclusion of alternative causes such as chronic kidney disease, myeloproliferative disorders and CMCD.

Chronic kidney disease is another common cause of elevated baseline serum tryptase therefore creatinine should be measured in any patient with hypertryptasemia [54, 58]. Patients with an elevated baseline tryptase should be questioned about weight loss, fever, nocturnal diaphoresis, examined for adenopathy and hepatosplenomegaly and a complete blood count (CBC) should be ordered as some hematologic neoplasia, most commonly of myeloid origin, can cause hypertryptasemia [54, 55]. It is currently thought that HαT—by increasing production of tryptase heterotetramers, which are more stable and vasoactive—acts as a disease modifier rather than a cause of MCA [59]. HαT is also more prevalent in patients with SM (around 15%) and IA (between 17 and 29%) compared to the general population [59].

The vast majority of CMCD associated with IA are caused by a somatic mutation (p.D816V) in *KIT* and a normal baseline serum tryptase does not rule out the possibility of a CMCD [19, 49, 55]. It is therefore recommended to order a KIT D816V mutation analysis on peripheral blood in all patients with IA regardless of the baseline serum tryptase value [49, 55]. The sensitivity of this assay to detect a mutation varies greatly depending on the technique used. It is recommended to use a highly sensitive assay with a detection capacity of at least 0.01% abnormal alleles such as an allele-specific quantitative PCR or a digital droplet PCR [60–62]. However, those high sensitivity tests are not readily available throughout Canada at present and may therefore require a send-out to a specialized laboratory if a less sensitive assay is negative and suspicion remains high [63].

Urine metabolites of mast cell mediators measured at baseline may also help predict mast cell clonality [39, 64]. Studies have shown that most patients with SM have elevated levels of NMH, 2,3dPGF2α, and LTE4 at baseline [39]. Therefore, whereas elevated levels of urine metabolites at baseline are not diagnostic of MCAS, they may increase the probability that the patient has a CMCD. In comparison, patients with HαT generally have normal levels at baseline despite an elevated baseline serum tryptase [64].

Despite the fact that most patients with SM presenting with IA have bone marrow mastocytosis without skin involvement, it is important to look for lesions suggestive of skin mastocytosis on physical examination followed, if present, by skin biopsy to confirm the diagnosis [55]. If suspicion of a CMCD is high (clonality prediction score ≥ 2), the criterion standard for diagnosing SM or MMAS is a bone marrow biopsy, which should ideally be performed in a center with expertise in SM since the burden of abnormal mast cells is typically quite low in those patients [49, 55, 65]. If KIT D816V was negative on peripheral blood, it is recommended to repeat the analysis on a bone marrow aspirated sample using a highly sensitive assay, which is considered the reference standard [60]. If a CMCD is identified, those patients should be considered at higher risk of severe recurrent anaphylaxis, especially from hymenoptera stings [49, 55]. Screening for venom sensitization (despite no history of a systemic venom reaction) and, if positive, venom immunotherapy should be discussed in patients with SM, according to the 2016 practice parameter update on stinging insect hypersensitivity, despite limited evidence [66, 67]. In addition, patients diagnosed with SM are at increased risk of developing osteoporosis and carry a very small risk of progressing to a more advanced subtype of SM [55]. Bone density surveillance over time and yearly CBC, liver function tests and baseline serum tryptase measurements are generally recommended [55].

## Management

A therapeutic response to medications capable of either blocking the effects of MC mediators or of stabilizing MCs or of reducing MC mediator production is a crucial diagnostic criterion for MCAS [3]. Self-administered epinephrine is the cornerstone treatment for acute episodes, especially those meeting criteria for anaphylaxis, given its rapid onset of action and efficacy [8, 27, 55]. Milder episodes may be adequately treated with oral antiH1 keeping in mind that they have a slow onset of action and are not as effective as epinephrine at treating a systemic MCA episode. Prophylactic treatment, typically starting with second-generation H1 antihistamines—which can be increased to 4 times the standard dose—should be discussed with patients with frequent (more than once or twice per year) or severe episodes, which may provoke significant anxiety [8, 11, 27, 55]. Thereafter, if symptomatic episodes with objective evidence of MCA persist, treatment can be individualized according to specific symptoms, using various combinations of medications that either target MC mediators or stabilize MC (Fig. 1). It should be noted that the evidence supporting current treatment options is very limited, relying primarily on expert opinion, extrapolation from their known mechanisms of action, and data from other conditions involving MCA. It may also be helpful to optimize treatment of coexisting conditions, such as chronic rhinitis or asthma, that may be exacerbated during MCA episodes by applying current treatment guidelines [68, 69].

1. H1-antihistamines (anti-H1)

H1 antihistamines are typically offered as initial treatment, as they can effectively alleviate cutaneous symptoms such as urticaria, angioedema, and histaminergic pruritus [8, 11, 19, 27, 55]. In a recent observational study involving 31 patients with idiopathic MCAS, all patients were on anti-H1—only a minority was on additional treatments—and all had a positive response [19]. In SM, several studies have shown a favourable response to anti-H1, including significant relief from urticaria, angioedema and pruritus [70–72]. A randomized, double-blind, placebo-controlled, cross-over trial of rupatadine 20 mg daily in adult mastocytosis patients showed significant improvement in quality of life (mainly attributable to reduced pruritus) while patients were on rupatadine compared to placebo [72]. Given the inferior side effect profile and lack of superior efficacy of first generation compared to second-generation anti-H1, the latter should be strongly prioritized over the former [73]. Depending on clinical circumstances, patients may be initiated on a standard dose, and then updosed if needed, or initiated at 4 times the standard dose, as in CSU [8, 11, 55]. In the absence of recurrent episodes, the dose can be gradually tapered, and the anti-H1 may eventually be discontinued, since as in CSU, some patients may enter remission [25].

2) H2-antihistamines (anti-H2)

H2 antihistamines are often empirically used as add-on therapy for patients experiencing GI symptoms associated with MCAS episodes [8, 11, 55]. Although they could theoritically provide symptomatic relief by blocking histamine-mediated acid secretion, this benefit has never been clearly demonstrated [8]. In addition, some evidence suggests that the combination of anti-H1 and anti-H2 is more effective than either alone in reducing certain cutaneous symptoms, such as pruritus and urticaria, during an acute allergic reaction [74, 75]. Therefore, given their favorable side effect profile, a trial is often attempted [8, 11, 55, 76].

3) Leukotriene receptor antagonists (LTRA)

In Canada, the only available LTRA option is montelukast. Despite the lack of studies exploring its efficacy in MCAS, a recent observational study of 31 patients with idiopathic MCAS reported that LTRA was the second most commonly used drug for symptom control after anti-H1 [19]. In support of its utility, a systematic review of 34 randomized controlled trials (RCT) in patients with urticaria showed a probable modest benefit of adding a LTRA to an anti-H1 on urticaria control [77]. Finally, given the marked increase in cysteinyl leukotrienes observed during episodes of systemic MCA, there may be a benefit in prophylactic receptor blockade [41, 55].

4) Sodium cromoglicate (cromolyn)

Oral cromolyn, which is the only formulation available in Canada, may be considered for individuals with persistent GI symptoms associated with MCAS episodes despite the use of anti-H1 and anti-H2 [78]. Although its mechanism of action is unknown, it is said to have MC stabilizing properties [55]. Evidence supporting its use mainly comes from a small randomized cross-over trial of 11 SM patients where it significantly alleviated symptoms of abdominal cramping and diarrhea compared to placebo [79]. In contrast, non-GI symptoms did not improve on cromolyn compared to placebo. It should be noted that patients selected for this study had previously taken cromolyn and were considered to be good responders thus introducing a selection bias. Oral cromolyn is minimally absorbed and is generally well tolerated [80]. Experts often suggest starting with a low dose and gradually increasing to the recommended dose of 200 mg four times daily [8]. It should be taken on an empty stomach before meals and at bedtime. Per product monograph, the preferred method of administration is to open the capsule(s) and put all of the powder in a cup, dissolve the powder in 1 teaspoonful of very hot water and dilute it with 4 teaspoonfuls of cold water [80]. Cromolyn should be taken for at least one month before judging efficacy [8]. Using oral cromolyn may be challenging in practice given its sometimes limited availability in pharmacies, difficulties in obtaining drug coverage in many parts of Canada as well as patient adherence to a drug that requires preparation and that is taken 4 times a day.

5) Ketotifen

Ketotifen is a first-generation anti-H1, more widely known for its mast cell-stabilizing property [81]. In a study involving 10 patients with SM, ketotifen dosed at 2 mg twice daily was shown to reduce symptoms of urticaria and pruritus compared to placebo, although 40% of participants experienced tiredness during treatment [82]. In a large case series of IA patients, addition of ketotifen reportedly allowed stopping prednisone in some patients that were considered corticosteroid-dependant [83]. However, in children with mastocytosis, ketotifen dosed at 1 mg two times daily was found to be less effective than hydroxyzine dosed at 2 mg/kg four times daily in controlling flushing and abdominal pain [70]. Nowadays, many experts suggest using ketotifen in patients who fail to be controlled with high-dose second generation antiH1 [8, 11, 55]. It should be introduced at a low dose and initially taken at bedtime due to its sedating effect. The recommended dose is up to 2 mg twice daily [84].

6) Aspirin

There is limited evidence that aspirin—by blocking the massive production of prostaglandin D2 that occurs with systemic MCA—may help prevent MCA episodes in patients with persistent episodes despite antiH1 [8, 41, 85]. The effective dose may vary from 80 to 650 mg twice daily [55, 86]. If NSAID tolerance is uncertain, an aspirin challenge should be considered before adding it to the treatment regimen. Due to the potential adverse effects of high-dose NSAID therapy, cardiovascular risk and renal function should be assessed, and gastroprotection with a proton pump inhibitor should be considered [87].

7) Oral corticosteroids

Prednisone in combination with anti-H1 has been used by some experts in patients with frequent episodes of IA (two episodes in the previous two months or 6 episodes in the previous year) [27, 88]. It can be started at a dose between 40 and 60 mg per day for 2 weeks and then gradually tapered by reducing the dose by 5 to 10 mg every 2 weeks [27]. Others opt for an alternate day regimen (dosing once every two days) once episodes have stopped and tapering by 10 to 20 mg every 1–2 weeks until a dose of 20 mg is reached and then by 5 mg every 1–2 weeks [88]. In a large case series dating from 1996, 56 patients had been treated with prednisone for recurrent IA for at least 3 months but very few were considered corticosteroid-dependant and required long-term therapy [83]. Given the known adverse effects of systemic corticosteroids, their use should be limited to severe cases refractory to the above-mentioned medications and, if tapering fails, omalizumab should be strongly considered [8, 27].

8) Omalizumab

For patients who experience severe recurrent reactions despite the aforementioned treatments, omalizumab may be considered as a treatment option [19, 27, 89–93]. While small RCTs in SM and IA have not demonstrated its efficacy, observational studies have often reported a significant reduction in anaphylaxis episodes after its initiation [89]. The RCT in IA—in which a trend towards less events in the group treated with omalizumab was detected—was probably underpowered (16 patients received at least one dose of either omalizumab or placebo) and of insufficient length (6 months) to detect a statistically significant difference [90]. Few patients were enrolled in the two RCTs in SM (30 and 16 patients, respectively) and none had anaphylaxis during the study period [91, 92]. Moreover, the use of a composite end-point combining 38 different symptoms, may not be adequate to capture the clinical benefit of omalizumab [89]. In contrast, Kaminsky et al. published a systematic review (which included the patients of the aforementioned RCT in IA) combined with a retrospective cohort of IA, and showed, that among 35 patients treated with omalizumab, 62% had a complete response, 29% had a partial response, and 9% were nonresponders [93]. However, the variable course of IA and the fact that many patients achieve remission without omalizumab limit interpretation [89, 93]. For MCAS, omalizumab is often used, off-label, at a dose of 300 mg every 4 weeks, as in CSU [89]. Only a small minority of MCAS patients will require a trial of omalizumab as illustrated in a recent case series where only 1 of 31 patients with idiopathic MCAS and 2 of 13 patients with clonal MCAS received it [19]. In Canada, accessing omalizumab through public or private insurance can be challenging, as the drug is currently approved only for CSU, allergic asthma, and chronic rhinosinusitis with nasal polyposis [94].

## Conclusions

Whereas MCAS may present significant diagnostic and therapeutic challenges, it should be emphasized that most patients can achieve adequate symptomatic control on anti-H1 and eventually experience disease remission, even without ongoing prophylactic treatment [19, 25, 83]. Given the level of anxiety that can be associated with this condition, clinicians should be attentive to signs of psychological distress, offer support, and discuss openly that anxiety is a normal response to stressful and unpredictable events such as spontaneous systemic MCA. In itself, anxiety may exacerbate symptomatic episodes and may even sometimes trigger episodes mimicking anaphylaxis [88].

At present, only a small minority of patients referred for suspected MCAS are ultimately diagnosed with the condition, underscoring the need for allergists to better communicate with the public and other medical professionals about which clinical presentations are consistent with MCAS and which ones are clearly not [19, 95]. Further research is needed to better define the clinical presentation, natural history, and treatment response as studies on MCAS remain scarce and original data from Canadian centers are lacking. Improving access to important diagnostic tools, such as urinary metabolites of MC mediators, sensitive PCR techniques for the KIT D816V mutation, and tryptase genotyping, may require developing these tests in Canadian laboratories. Looking ahead, remibrutinib and monoclonal antibodies targeting mast cells hold promise for MCAS management. Addressing these challenges will require ongoing research, refining diagnostic criteria, and enhanced education and resources for healthcare providers to better support patients navigating this complex and often misunderstood condition.

## Data Availability

No datasets were generated or analysed during the current study.

## Abbreviations

2,3dPF2α: 2,3-Dinor 11β-Prostaglandin F2α
Anti-H1: H1-antihistamine
Anti-H2: H2-antihistamine
CSU: Chronic spontaneous urticaria
CMCD: Clonal mast cell disorder
CBC: Complete blood count
ECNM-AIM: European Competence Network on Mastocytosis-American Initiative on Mast Cell Diseases
GI: Gastro-intestinal
HαT: Hereditary alpha-tryptasemia
IA: Idiopathic anaphylaxis
LTE4: Leukotriene E4
LTRA: Leukotriene receptor antagonist
MC: Mast cell
MCA: Mast cell activation
MCAS: Mast cell activation syndrome
MMAS: Monoclonal mast cell activation syndrome
NICAS: National Institute of Health Idiopathic Clonal Anaphylaxis Analysis Score
NMH: N-methylhistamine
NSAID: Nonsteroidal anti-inflammatory drug
RCT: Randomized controlled trial
REMA: Spanish Network on Mastocytosis
SM: Systemic mastocytosis
